# Characteristics of 3D Printable Bronze PLA-Based Filament Composites for Gaskets

**DOI:** 10.3390/ma14164770

**Published:** 2021-08-23

**Authors:** Marcela Sava, Ramona Nagy, Karoly Menyhardt

**Affiliations:** Department of Mechanics and Strength of Materials, Politehnica University Timisoara, 2 Victoriei Sq., 300006 Timisoara, Romania

**Keywords:** composite, PLA, bronze, 3D printing, gaskets

## Abstract

Composite materials can be tailored for various properties, but the manufacturing process can be quite lengthy depending on the complexity of the final product. Instead, we focused our attention on the relatively new technology of additive manufacturing (3D printing) that can produce complex geometries for a limited number of samples. Due to the weak bond between successive printed layers, these objects will have weaker mechanical properties in relation to cast or sintered materials. Thus, the orientation of the printed layers can make a huge difference in the behavior of the products. In this paper, a 3D printed composite made from bronze-filled PLA is mechanically characterized in order to be used as a substitute for sintered compacted bronze products for compression loads. Thus, cylindrical samples grown with the base horizontally and vertically were subjected to compression loads to determine their stress-strain curves at room temperature as well as in the glass transition region. Due to a lack of published research in this area, this study offers an insight into the usability of bronze-filled PLA for gaskets or other objects subjected to compression loads.

## 1. Introduction

Composite materials have played an important role throughout human history, from housing early civilizations to enabling future innovations. Composites offer many benefits like corrosion resistance, design flexibility, durability, strength, and they are lightweight. The weight savings translates into energy savings or increased performance [1,2].

Composites can be tailored for various properties by appropriately choosing their components, proportions, distributions, morphologies, degrees of crystallinity, crystallographic textures, as well as the structure and composition of the interface between components. They have permeated our everyday lives, such as products that are used in constructions, medical applications [3], automotive [4,5,6,7,8] and electronics industry [9], aerospace, defense [10], oil and gas transportation, sports, and many more. 

The composites industry is a constantly evolving one, working with new materials; processes and applications are being developed all the time—like using hybrid virgin and recycled fibers, faster and more automated manufacturing. As a result, composite materials constitute most commercial engineering materials. The global composites materials market is growing every year. Metal matrix composites represent 10% of the production of composite materials, as matrix metals are chosen Al, Ti, Mg, Ni, Cu, Fe, [11,12]. In the last decades, specific processes of powder metallurgy were elaborated for composites materials [13].

There are a lot of composites which are manufactured by additive manufacturing. Additive manufacturing is playing an increasingly important role in the manufacturing industry, is mainly used in toolmaking and prototype construction, and enables the creation of 3D objects. Additive manufacturing includes processes in which the part to be produced is constructed by the addition of material. The construction is carried out in layers [14]. There are several types of 3D printing processes, of which Fused Deposition Modelling (FDM) technology is one of the simplest and most cost-effective [15]. The FDM process involves layering materials like plastics, composites, or biomaterials to create objects that range in shape, size, rigidity, and color. The suitability and implementation of 3D-Printing for small-scale series production are presented and discussed by some authors [16,17,18,19]. The resulting strength, stiffness, and rigidity of printed objects are considered limiting factors for the more widespread adoption of FDM.

One of the most common raw materials in the FDM process is PLA polylactic acid. PLA is thermoplastic semi-crystalline polyester, a biopolymer that can be made from natural materials such as starch and sugar, and it can be decomposed by composting under industrial conditions [20,21]. Nowadays, environmental protection is very important, so biopolymers such as PLA have become the center of interest [22,23]. The elevated environmental awareness and the good properties (high tensile strength and Young’s modulus, good flexural strength) have resulted in the expanded use of PLA for consumer goods and packaging applications. Furthermore, it is expected that novel technological advances will lead to a boom in the biopolymer market in the transportation and automotive industries Due to its high mechanical strength and good processability, it has great potential to replace conventional materials; composite PLA can be used [24].

The purpose of this work is the study of the mechanical properties of the bronze-based PLA, mainly for compression loads and the comparison between the mechanical properties of PLA bronze-filled printed samples, and CuSn10 sintered bronze powder samples.

One of the many uses of bronze due to its good corrosion resistance and hardness is for gaskets. Gaskets are flat seals that can be made in any shape required. They can be made from a range of materials, including paper, rubber, silicone, leather, metal, cork, felt, neoprene, nitrile rubber, fiberglass, polytetrafluoroethylene, polymers and are often made with layers of different materials. Non-asbestos gasket sheets are made using organic fibers, aramid fibers, NBR (nitrile butadiene rubber), and mineral fibers. These materials can be used for applications where water or oil resistance is required. 

**Figure 1:**
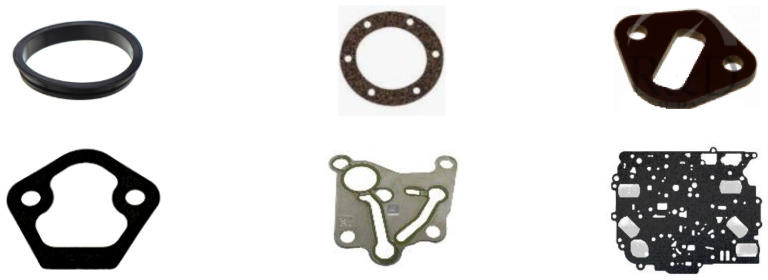
Selection of gasket geometry, from simple to complex.

Gaskets are usually used for sealing elements in all kinds of hydraulic pumps. A fuel pump gasket is a gasket, or a piece of flexible material intended to prevent leakage, that is positioned between a mechanical fuel pump and the mounting surface on the side of the engine. The fuel pump gasket prevents the engine oil from leaking out. A gasket, Figure 1, therefore, must be flexible enough to fill all the spaces and still compress when needed, but also strong enough to withstand the temperatures of an engine and its components [25].

The geometry of gaskets can vary from simple circular forms to complex shapes that need expansive molds and dies. A more cost-effective way to produce these gaskets is to use 3D printing technology that can create custom gaskets in a relatively short time.

Having little to none published papers regarding FDM printed bronze-filled PLA gaskets under compression loads, this paper brings new and important data concerning the behavior of the materials. Thus, in this paper, we present some characteristic parameters for FDM 3D printed bronze-filled PLA that can substitute classical bronze alloys for complex shaped gaskets. The study of the mechanical behavior of cold-pressed powders is important to predict the response of the powder material in terms of stresses and strains [26,27,28,29].

## 2. Materials and Methods

**Figure 2:**
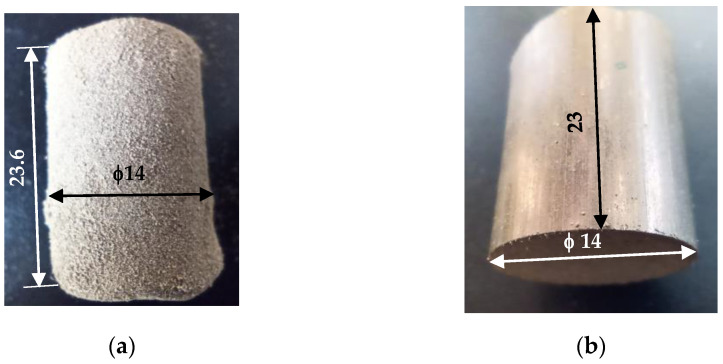
Bronze powder samples: (**a**) green compact, (**b**) sintered compact.

In the past, alternative methods were used to obtain bronze gaskets, starting from sintered compacted bronze powder (Figure 2). 

The behavior of an industrial bronze powder (CuSn10) was studied during compression, before and after the sintering process, for its experimental characterization. A constant amount of bronze powder (24 g) was pressed in a rigid steel alloy mold with forces of 80 kN to obtain cold die-pressed compacts (“green compacts”) of 23 mm height, as shown in Figure 2a. The rigid cylindrical steel alloy mold’s height is 70 mm, and its interior diameter is 14 mm. The mold, placed in the compression test machine jaws, imposes zero radial strain of the powder grains during compression. Some cold die-pressed compacts were submitted to different sintering processes (temperatures of 725 °C, 775 °C, 825 °C, and 15–120 min hold time) to improve the structure and the mechanical characteristics. The sintering process was performed in an Argon atmosphere to avoid the oxidation of the samples [30,31]. 

The composite material used in the following study is MetalFil-Ancient bronze, a PLA-based filament with approximately 80% bronze powder. Table 1 shows the MetalFil’s physical properties provided by the manufacturer [32,33].

As is shown in Table 1, the compression strength and compressive modulus are not provided by the manufacturer and had to be determined.

**Figure 3:**
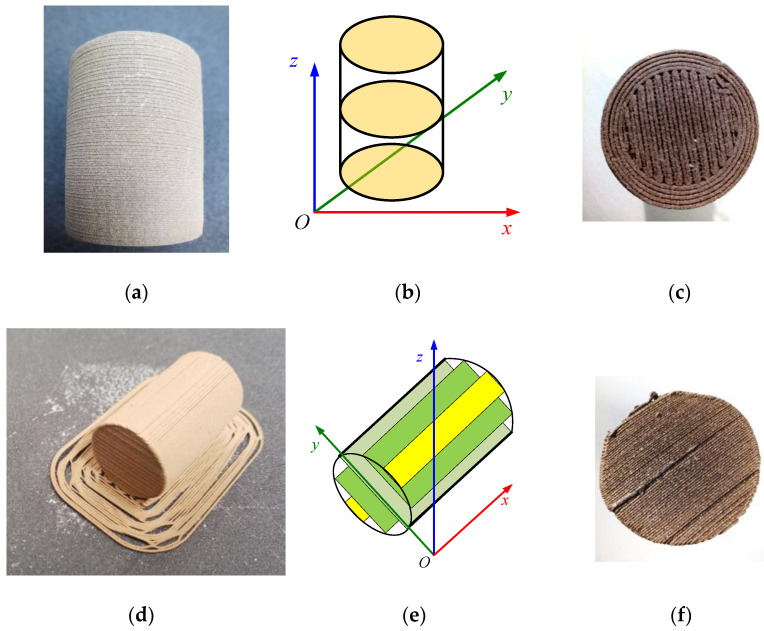
PLA bronze-filled samples: (**a**) vertically layered printed sample; (**b**) schematic of vertically layered printed sample; (**c**) top view of the vertically layered printed sample; (**d**) horizontally layered printed sample; (**e**) schematic of horizontally layered printed sample, (**f**) top view of the horizontally layered printed sample.

Considering the compression load, two FDM building methods were considered to obtain the test samples (Figure 3): one having vertical layers (a, b, c) and the other having horizontal layers (d, e, f), referenced to the base of the cylinder and it is positioning on the printer table during the printing process.

The first sample type was built with the basis of the cylinder horizontally (horizontal layers/vertically layered), as shown in Figure 3b. The second sample type was built by rotating the cylinder by 90 degrees; thus, the circular base was in a vertical plane (vertical layers/horizontally layered) as shown in Figure 3e. These two configurations were considered the only logical approaches to 3D printer building. The sample parts (stl files) were designed in FreeCad and converted to G-code in Ultimaker Cura.

As required by standard [34,35], five test samples were used for each building type, having 14 mm in diameter and 23 mm in height, as the sintered bronze powder ones. The PLA bronze samples were printed on a Creality CR-10 MAX printer (Shenzhen Creality 3D Technology Co., Shenzhen, China), having a layer thickness of 0.1 mm, 0.4 mm nozzle, 100% fill at 220 °C. In additive printing, the main process parameters that directly influence the quality of the products are ambient temperatures, layer thickness, the geometry of the part, position and orientation, material type and quality [36].

In order to determine the mechanical properties, the printed samples were subjected to a simple compression test. The samples were tested on an Instron 8800 biaxial servo hydraulic fatigue testing system (Instron, Norfolk County, MA, USA), controlled in displacement for 10 mm.

For high-pressure hydraulic equipment, the hydraulic oil pressure is up to 400–600 bars, and the temperature is around 80 °C. For medium pressure hydraulic equipment, the hydraulic oil pressure is around 200 bars, and the temperature is 40–50 °C. This is the case for construction machinery, where the pressure is set by the manufacturer, and the temperature is indicated by the equipment. The printed samples were immersed for 48 h in gasoline, diesel, and synthetic transmission oil; they were weighed before and after immersion showing the same mass.

The densities of the samples were determined using a Kern PRJ 620-3M precision balance (Kern & Sohn, Albstadt, Germany), with a 0.001 g precision, measuring the mass of the samples in air and water.

## 3. Results and Discussions

**Figure 4:**
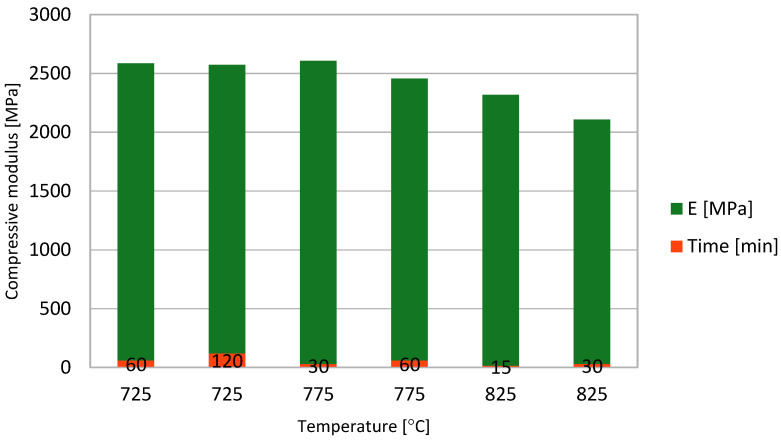
Compressive modulus of the sintered powder bronze samples versus sintering time and temperature.

Using the results obtained by Sava in [31] for sintered bronze powder samples with densities ranging between 7–7.4 g/cm^3^, the elasticity modulus values for different sintering processes were calculated and are shown in Figure 4. The compression elasticity modulus of sintered bronze powder samples decreases with an increase in sintering temperature and sintering time.

In Figure 4, each green column represents the compressive modulus obtained at various sintering temperatures (725–825 °C), and each red column is the hold time of the sintering process.

**Figure 5:**
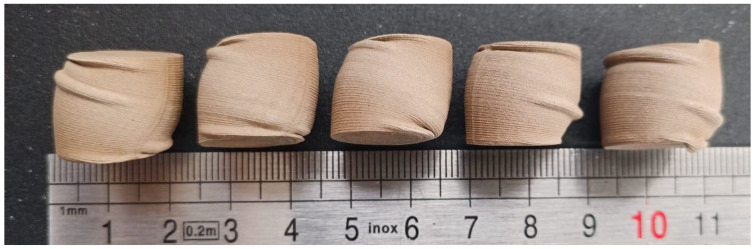
Vertically layered printed samples after compression load.

**Figure 6:**
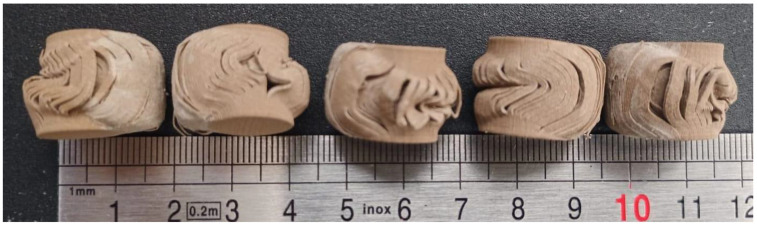
Horizontally layered printed samples after compression load.

Figure 5 shows the resulting vertically layered printed deformed specimens, and Figure 6 shows the resulting horizontally layered printed deformed specimens.

The resulting deformed specimens after approximately 10 mm displacement show a barrel shape of the samples after compression (Figure 5 and Figure 6). As can be observed, layers tend to slide, and if clearance is not allowed, layers breaking occurs. 

Considering the compressive load F and the area of original cross-section A, the compressive stress can be calculated [37]:

(1) $$\sigma =\frac{F}{A}$$

The compressive strain can be determined as the ratio between the decrease in sample length Δl and initial length l_0_ of the test specimen [35]:

(2) $$\varepsilon =\frac{\Delta l}{l_{0}}$$

**Figure 7:**
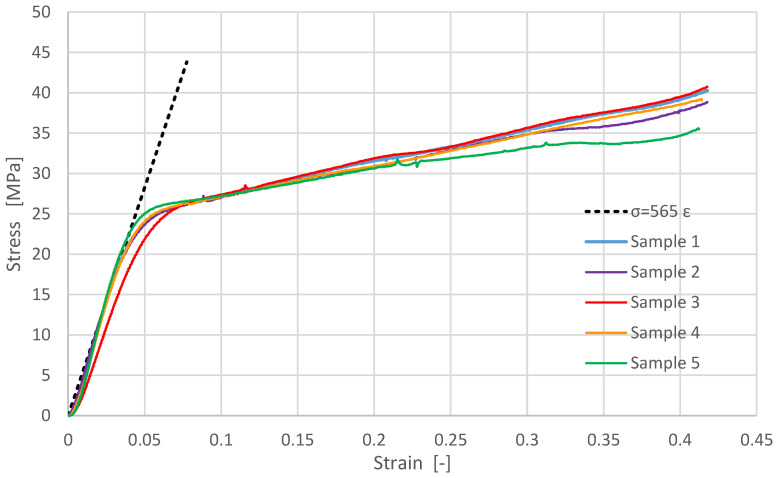
Experimental results for vertically layered printed samples: engineering compressive stress versus compressive strain.

Figure 7 shows the engineering compressive stress versus the sample compressive strain of the vertically layered samples. The dotted line represents the tangent to the stress-strain curves in the elastic domain.

**Figure 8:**
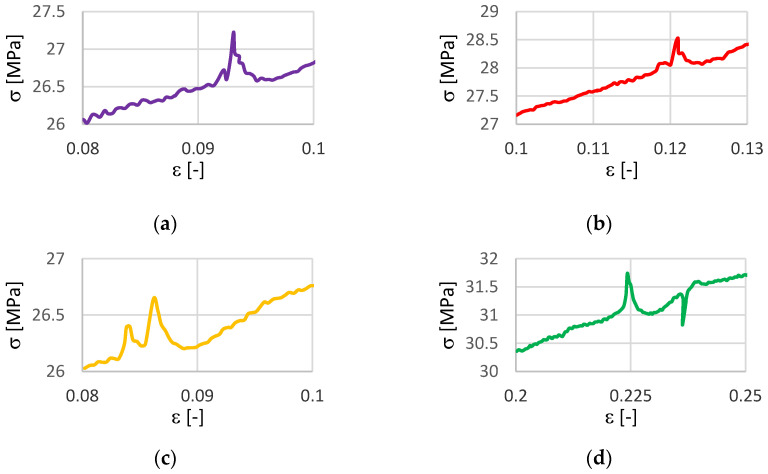
Details from Figure 7: (**a**) for sample 2; (**b**) for sample 3; (**c**) for sample 4; (**d**) for sample 5.

The testing results for samples 2–5 show stress peaks for stresses between 26–32 MPa, due to the inhomogeneity of the vertically layered printed samples. Figure 8 shows details from Figure 7 for samples 2–5.

**Figure 9:**
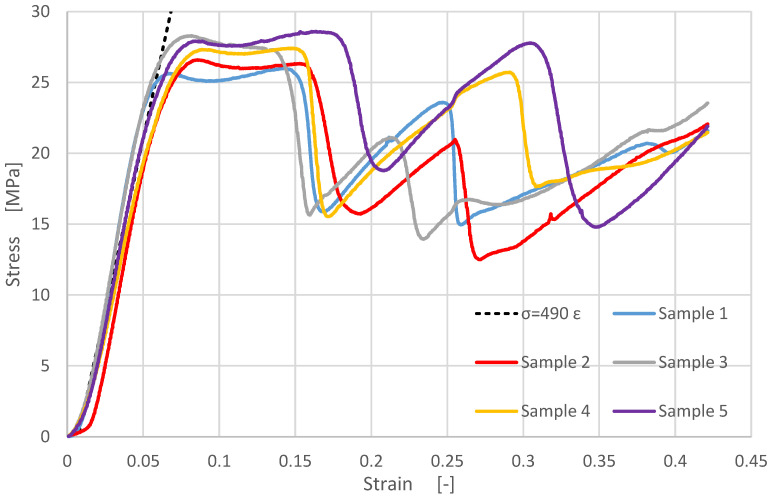
Experimental results for horizontally layered printed samples: engineering compressive stress versus compressive strain.

For the horizontally layered samples, the tests were performed similarly, resulting in a jagged curve (Figure 9) denoting the breakage in layer binding. The dotted line represents the tangent to the stress-strain curves in the elastic domain.

In order to better understand the elastic behavior of gaskets made from this material, an in-depth analysis was performed to verify and quantify the elastic modulus. As is well known, the modulus of elasticity characterizes the test specimen’s resistance to elastic (reversible) deformation, being the slope of the stress-strain curve in the elastic deformation field [37]:

(3) $$E=\frac{\sigma }{\varepsilon }$$

where *E* is the compressive modulus, σ is the compressive stress, and ε is the compressive strain. 

Experimentally, the compressive modulus was determined as the ratio of the stress difference to the corresponding strain difference values. Considering the slopes from Figure 7 and Figure 9, the compressive elastic modulus was determined for both building types, resulting in: compressive modulus of the vertically layered printed samples E_V_ = 565 MPa and compressive modulus of the horizontally layered printed samples E_H_ = 490 MPa.

**Figure 10:**
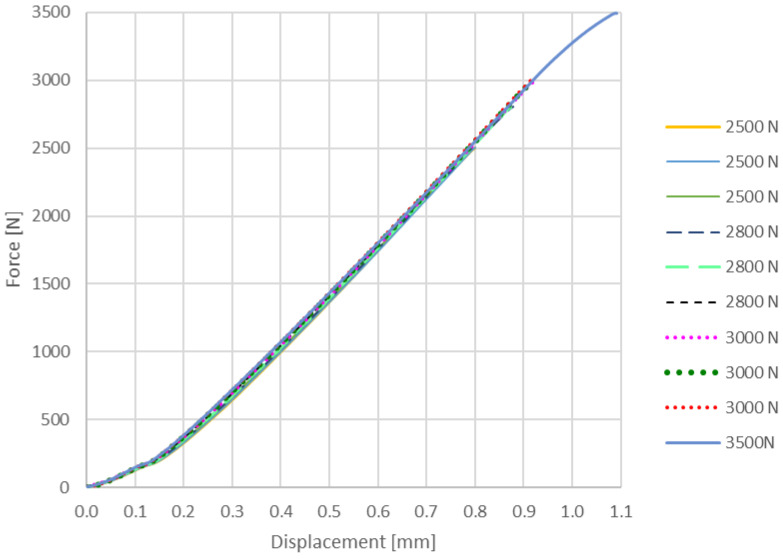
Proof of compression elastic range up to 3000 N for vertically layered samples.

**Figure 11:**
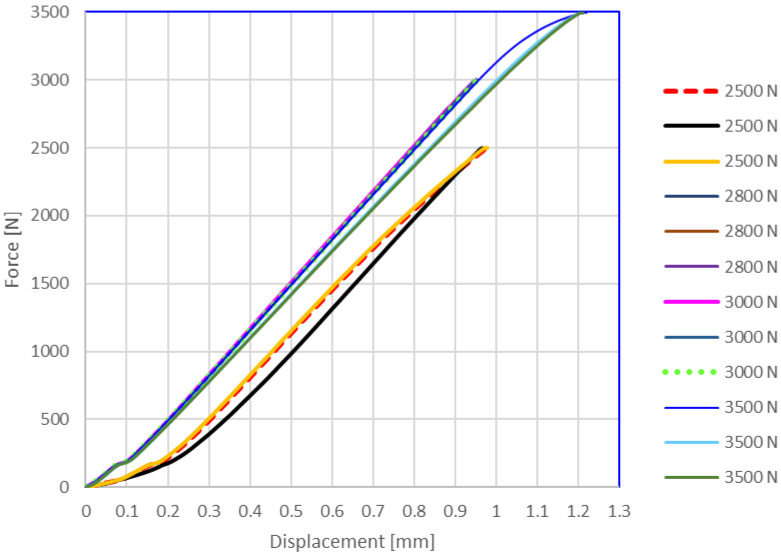
Proof of compression elastic range up to 3000 N for horizontally layered samples.

After the compressive modulus determination, each sample was subjected to cyclic loads to analyze the behavior in the elastic domain. These tests are shown in Figure 10 and Figure 11, being useful to limit the clamping force for gaskets made from this composite material. The elastic behavior of the samples in the 0–3000 N range can be noticed, confirming the repeatability of the phenomena for the vertically layered samples.

The glass transition temperature (T_g_) of amorphous PLA lies between 55 to 60 °C and is a function of the PLA molecular weight and stereochemistry. In semi-crystalline PLA, the Tg is higher (60–80 °C) and depends on the crystallization conditions that determine both the morphology of the crystalline/amorphous phases and the degree of crystallinity [38]. In order to assess the behavior of the samples in the glass transition temperature domain, samples were tested at 40 °C, 60 °C, and 80 °C. 

**Figure 12:**
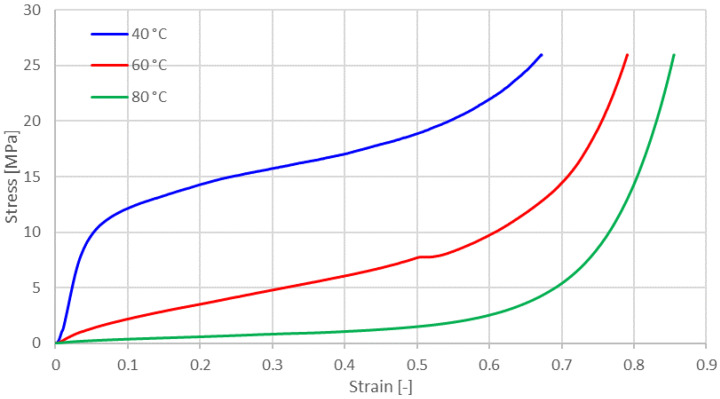
Experimental results for vertically layered printed samples: engineering compressive stress versus compressive strain at different temperatures.

Figure 12 shows the experimental results for vertically layered printed samples engineering compressive stress versus compressive strain at different temperatures, and Table 2 shows the samples measured density after compression at various temperatures. All samples have the same density at the beginning of the tests (3.29 g/cm^3^).

Comparing the data from Figure 4, Figure 7, Figure 9 and Figure 12, depending on the acting loads, one can choose the appropriate material to be used. Table 3 shows a succinct comparison between FDM 3D printed MetalFil-Ancient bronze and 60 min sintered bronze powder at 725 °C respectively at 775 °C.

## 4. Conclusions

Throughout this paper, mechanical characterization is made for bronze powder enriched polylactic acid material in relation with sintered bronze powder. Bronze-filled PLA gaskets are easier to manufacture in comparison with sintered compacted bronze and are more malleable.

Due to the nature of the additive manufacturing process, a novel and cost-effective manufacturing alternative, both vertical and horizontal layered samples were considered and presented.

This study presents guideline values for the correct and nondestructive usage of PLA bronze-filled gaskets or similar items created from this type of material submitted to compression loads.

In the lack of mechanical properties offered by the manufacturers of 3D printing filaments, which is understandable considering the multitude of post-processing variables involved in 3D printing, a unique study was performed, and the results were presented. The density of the printed sample (3.29 g/cm^3^) differs from that given by the filament manufacturer (3.5 g/cm^3^) even though it has a 100% fill printing setting. This value may also vary depending on the geometry and nozzle size of the printed sample. The compressive modulus for the MetalFil-Ancient bronze was determined in order to be used for gasket design.

Considering the bounding material PLA, printed bronze-filled PLA has stable mechanical properties only for working temperatures below 40 °C.

For a pressure load less than 19.48, MPa bronze-filled PLA is a suitable substitute for bronze gaskets working at temperatures below 40 °C.

With new materials being discovered and developed, it is possible to combine the advantages from both classical bronze sintered powder and bronze-filled PLA materials. These processes use specialized filament with more than 90% bronze and filler. The specimen can be printed on any FDM printer. Depending on the filler type, a debinding process of the specimen may be needed; afterwards, it will be sintered at a temperature of around 850–900 °C, resulting in stable mechanical properties at higher temperatures than those of PLA based materials [39].

## Figures and Tables

**Table 1:** Typical Material Properties of MetalFil-Ancient bronze, PLA-based filament.

Physical Properties	Unit	Value	Method
Specific gravity	g/cm^3^	3.5	ISO 1183
Melting temperature	°C	200 ± 10	ISO 294
Tensile Strength	MPa	19	ISO 527
Tensile Modulus	MPa	3990	ISO 527
Elongation @ break	%	8	ISO 527
Impact Strength (Izod-Un 23 °C)	kJ/m^2^	11.3	ISO 179
Soluble in water	Insoluble		
Hazardous reactions	The product is chemically stable		

**Table 2:** Samples density versus temperature at 3000 N load for vertically layered printed samples.

Density [g/cm^3^]	Load Temperature [°C]	Resulting Sample
3.29	24	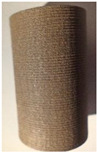
3.44	40	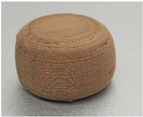
3.49	60	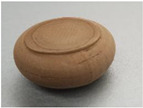
3.74	80	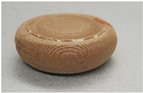

**Table 3:** Compressive modulus comparison between FDM 3D printed bronze-filled PLA and 60 min sintered bronze powder.

Material	Density [g/cm^3^]	Compressive Modulus [MPa]	Maximum Working Temperatures [°C]	Reference
MetalFil—Ancient bronze Vertically layered	3.29	565	+ 40	This work
MetalFil—Ancient bronze Horizontally layered	3.29	490	+ 40	This work
725 °C Sintered bronze powder	7.1	2526	+ 90	[31]
775 °C Sintered bronze powder	7.2	2397	+ 90	[31]
